# IgG4-related intracranial hypertrophic pachymeningitis with skull hyperostosis: a case report

**DOI:** 10.1186/1471-2482-13-37

**Published:** 2013-09-21

**Authors:** Che-Kuang Lin, Dar-Ming Lai

**Affiliations:** 1 Division of Neurosurgery, Department of Surgery, Far Eastern Memorial Hospital, No. 21, Sec. 2, Nanya S. Rd., Banciao Dist., New Taipei City 220, Taiwan; 2 Division of Neurosurgery, Department of Surgery, National Taiwan University College of Medicine and National Taiwan University Hospital, No. 7, Chung Shan S. Rd., 10002 Zhongzheng Dist., Taipei City, Taiwan

**Keywords:** IgG4, Intracranial, Pachymeningitis, Hyperostosis

## Abstract

**Background:**

Immunoglobulin G4 (IgG4)-related disease is a systemic syndrome, characterized by sclerosing lesions and usually associated with a raised serum IgG4 level; the pancreas, salivary glands, and lacrimal glands are typically affected. Recently, it has been suggested that IgG4-related sclerosing disease represents a subset of cases previously diagnosed as idiopathic hypertrophic pachymeningitis. This rare inflammatory disorder causes localized or diffused thickening of intracranial dura mater. Headache, cranial nerve palsy, and ataxia are the most common clinical manifestations.

Herein, we report the clinical and histopathological features of a rare case of IgG4-related intracranial hypertrophic pachymeningitis involving cranial hyperostosis.

**Case presentation:**

A 52-year-old man presented with refractory generalized tonic-clonic seizure. Magnetic resonance imaging revealed thickening of the meninges with enhancement near the superior sagittal sinus; skull bone defect was also noted. Extensive excision of affected skull bone and dura was performed, providing the diagnosis of IgG4-related pachymeningitis. After the surgery, the patient’s seizure stopped and he was smoothly tapered off antiepileptic medication.

**Conclusion:**

To our knowledge, this is the first reported case of IgG4-related pachymeningitis with concomitant skull hyperostosis.

## Background

Immunoglobulin G4 (IgG4)-related disease is a systemic syndrome, characterized by sclerosing lesions and usually associated with a raised serum IgG4 level [1]; the pancreas, salivary glands, and lacrimal glands are typically affected. Recently, it has been suggested that IgG4-related sclerosing disease represents a subset of cases previously diagnosed as idiopathic hypertrophic pachymeningitis [2-4]. This rare inflammatory disorder causes localized or diffused thickening of intracranial dura mater [5]. Headache, cranial nerve palsy, and ataxia are the most common clinical manifestations.

Herein, we report the clinical and histopathological features of a rare case of IgG4-related intracranial hypertrophic pachymeningitis involving cranial hyperostosis.

## Case report

A 52-year-old man with a history of treated hypertension and diabetes mellitus presented to our hospital following a single seizure attack; he regained consciousness shortly before arrival. Two weeks before this, he had a head injury that resulted in some minor scalp abrasion and bleeding. No obvious neurological deficit was noted at that time, and he was discharged following a period of observation without imaging. However, on the day after the discharge, he experienced another seizure attack that lasted for 5–6 minutes with subsequent loss of consciousness. He was then admitted to our hospital.

Initially, the patient experienced generalized tonic-clonic seizures twice daily. Persistent right calf paresthesia was also noted; this presented as a focal seizure when aggravated and often progressed to a generalized tonic-clonic seizure. Under antiepileptic medications, focal seizures remained persistent but progression to secondary generalized seizures became rare.

On physical examination there were no clinical signs to suggest other organ involvement, in particular there was no glandular enlargement or orbital disease. Abdominal ultrasound showed a normal liver, gallbladder, pancreas and kidneys.

**Figure 1:**
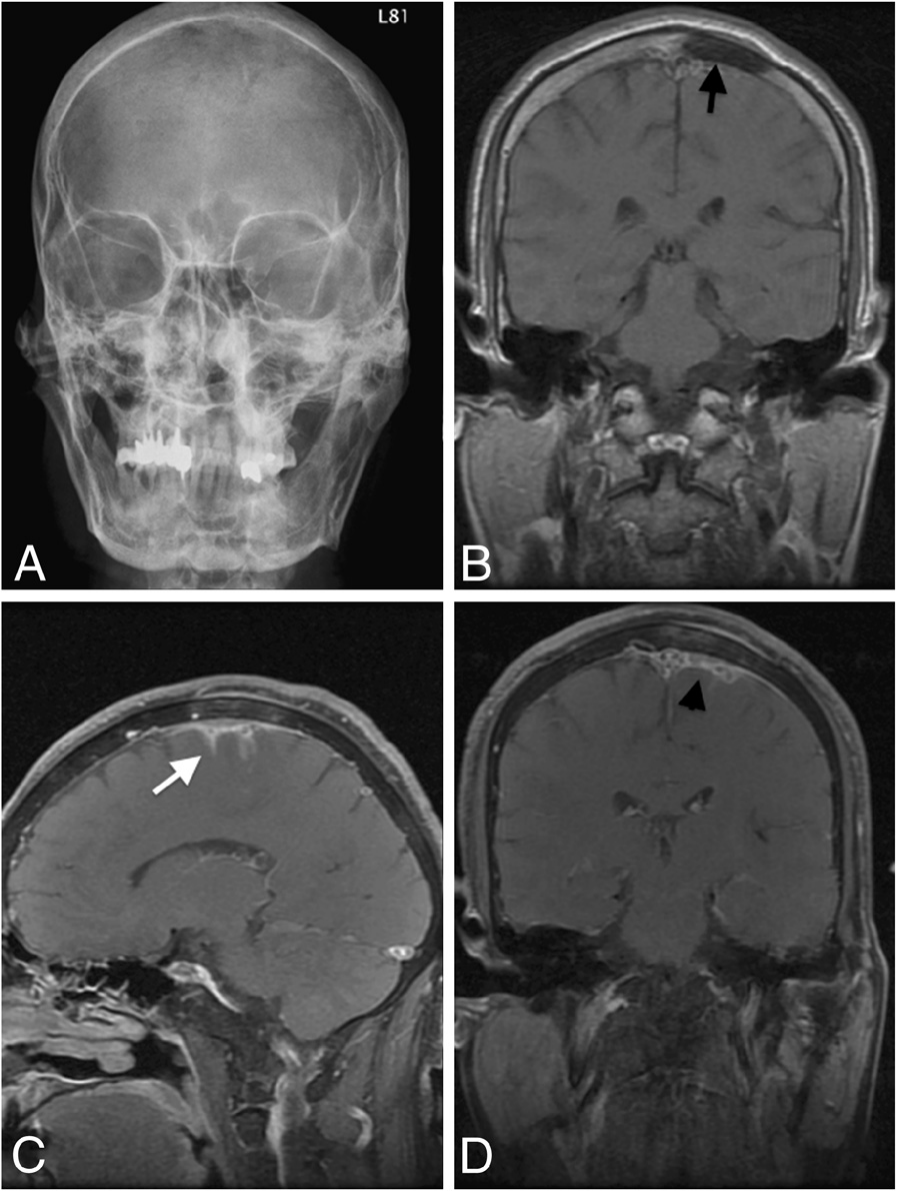
**Preoperative images. A.** Anteroposterior view of skull X-ray radiography shows left skull bone effacement near the vertex. **B-D**. Magnetic resonance image shows an area of abnormal bony marrow over left high convexity showing loss of normal fatty marrow with moderate enhancement after contrast injection (arrow). Mild subperiosteal soft tissue thickening and adjacent dura and leptomeningeal thickening and enhancement (arrowhead) are apparent. The left central and precentral sulci are filled with abnormal enhancing lesion (white arrows).

Magnetic resonance imaging showed thickening of the meninges with enhancement near the superior sagittal sinus; skull bone defect was also noted (Figure 1A-D). We initially suspected meningitis or skull metastasis with dura invasion. Subsequently, lumbar puncture was performed for cerebrospinal fluid (CSF) examination. Sugar level was within normal limit compared to peripheral blood sugar levels, and no visible leukocytes were noted. Hemogram did not reveal leukocytosis, and C-reactive protein level was also within the normal limit. Thus, infection seemed unlikely. CSF cytology revealed no malignant cells.

**Figure 2:**
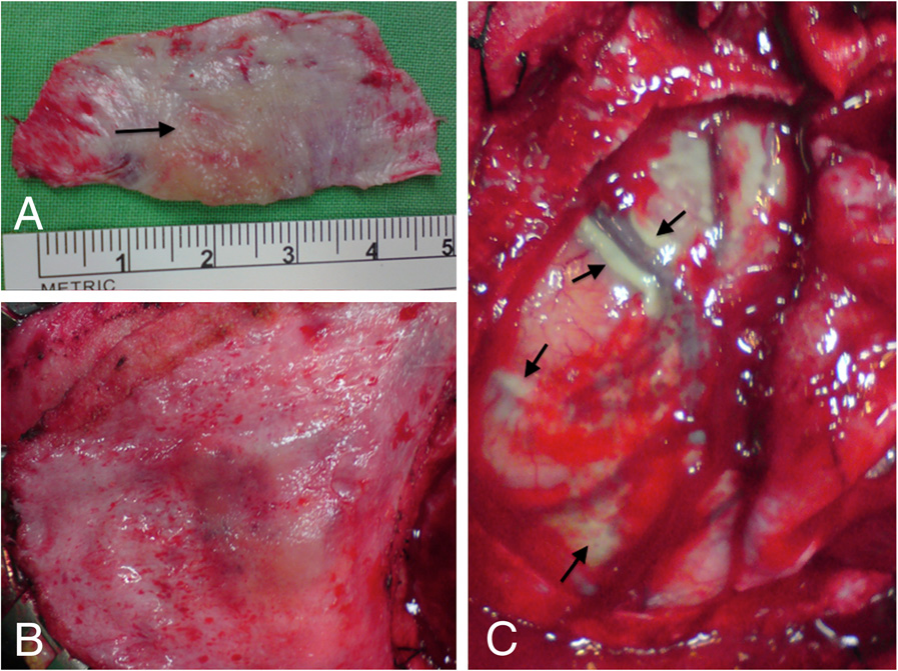
**Intraoperative images. A.** The dura with nodule at the central part (arrow). **B**. Galeal thickening. **C**. The secondary dural change along the cortical vessels and gyrus is noted just below the involved dura area (arrows).

Left parietal skull and dural nodule excision were then performed for both pathological diagnosis and symptomatic relief (Figure 2A). Duroplasty was also performed with autologous fascia, and the skull defect was covered with Titanium mesh. Hyperostosis of the skull was noted, and the overlying galea aponeurotica showed thickening and a whitish color change (Figure 2B). Gliosis was noted along the cortical vessels and gyrus just below the involved dura area (Figure 2C).

**Figure 3:**
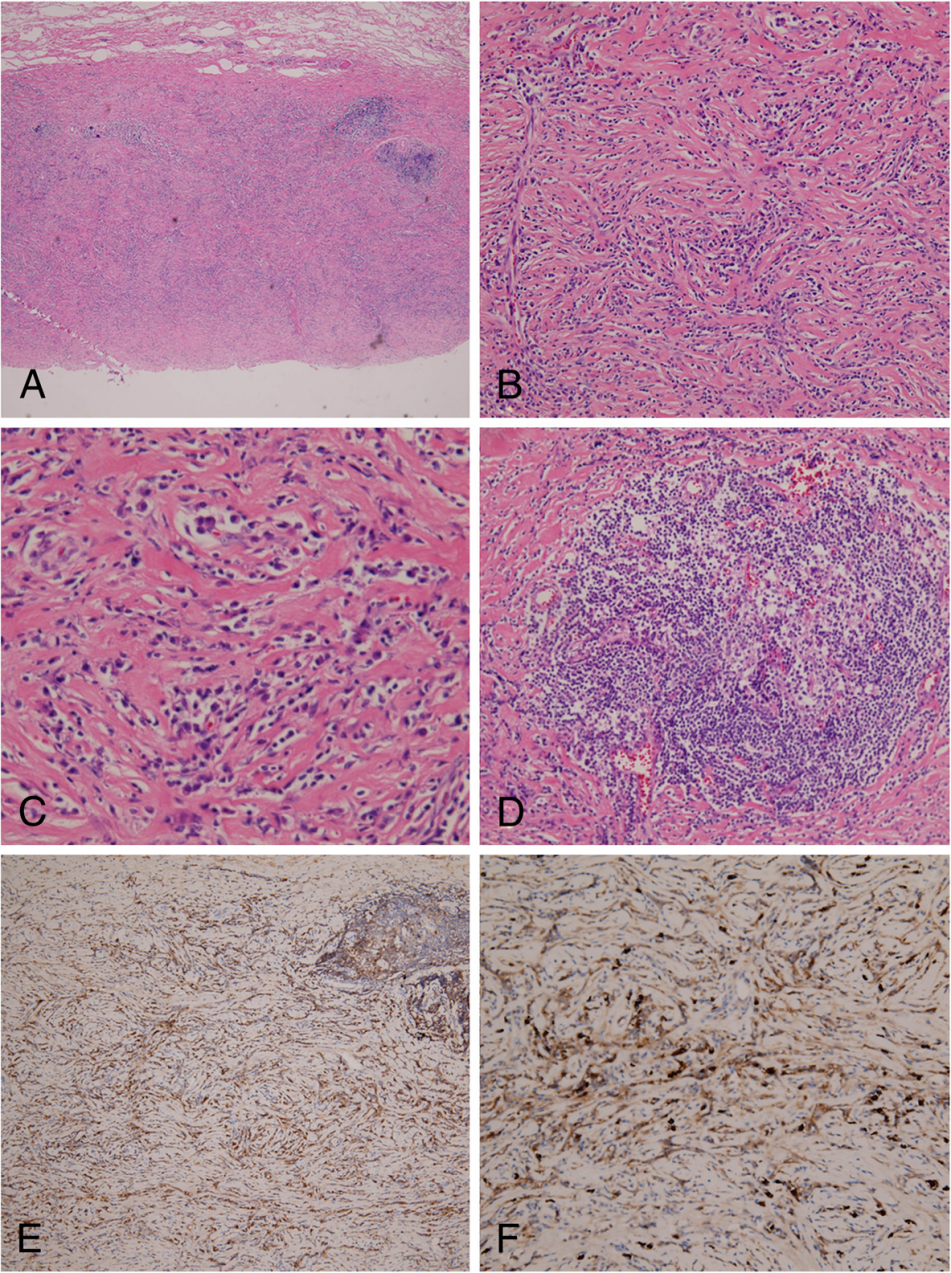
**Histopathological findings. A-D.** Sections of the dura and galea show increasing inflammatory change (**A, H&E**, ×40) composed of mainly lymphoplasma cells in a storiform fibrous background with occasional eosinophils (**B, H&E**, ×100; **C, H&E**, ×200). Lymphoid follicles with germinal center formation are also seen in a section of galeal tissues (**D**, **H&E**, ×100). **E&F**. IgG4 (+) plasma cells account for more than 50% of IgG (+) cells with a mean more than 30 in 3 higher power fields (ABC immunohistochemical stain, **E**, ×100; **F**, ×200).

Microscopically, sections of the dura and galea showed mixed inflammatory cell infiltration, composed mainly of lymphoplasma cells, in a stroiform fibrous background with occasional eosionphils. Lymphoid follicles with germinal center formations were also observed in the galeal tissue sections (Figure 3A-C). Skull sections revealed focal fibrosis and chronic inflammation in the marrow space. Immunohistochemical analysis revealed some inflammatory cells and spindle cells that were immunoreactive to S-100 stain. We concluded that a chronic inflammatory process was occurring, and a diagnosis of idiopathic hypertrophic cranial pachymeningitis was subsequently considered.

Immunohistochemical analysis using the avidin-biotin-complex (ABC) stain revealed an increased IgG4 (+) plasma cell count, which accounted for more than 50% of the IgG (+) cells with a mean more than 30 in 3 high power fields (HPFs); this was assessed by counting 20 HPFs [6] (Figure 3D & E). No evidence of spirochete infection was observed. Autoimmune profiles were normal, including findings for antinuclear antibody and rheumatoid factors. Increased serum IgG4 (939 mg/dl; normal ranges: 1–140 mg/dl) [1] was noted. Based on the histopathological and immunohistochemical findings, as well as the exclusion of known causes of pachymeningitis, a diagnosis of IgG4-related hypertrophic intracranial pachymeningitis was made.

After total excision of the affected skull bone and meninges, the patient’s seizures stopped; he was smoothly tapered off antiepileptic medication. In addition, his serum IgG4 levels returned to normal, and no obvious sequela was noted after the operation. Follow-up imaging study and physical examinations in 3 months later revealed that the infiltration of the arachnoid membrane had vanished and there was no involvement of other organs.

## Discussion

Hypertrophic pachymeningitis is a rare disorder that causes localized or diffused thickening of the dura mater [5]. Numerous clinico-pathological entities cause thickening of the pachymeninges, thus idiopathic hypertrophic pachymeningitis is diagnosed by exclusion; a dural biopsy is usually essential for a definitive diagnosis [5,7]. Pathological findings consist of thick fibrous dura often associated with chronic inflammation cell infiltrate consisting of lymphocytes and plasma cells; compression of neural structures by the thickened fibrous dura results in neurological defects. Hypertrophic cranial pachymeningitis most commonly presents with headache and cranial neuropathies [7]. Other associations include seizure, memory loss, ataxia, Tolosa-Hunt syndrome, and pituitary dysfunction [7,8]. The condition has also been described in relation to specific infections, including Lyme disease, syphilis, Mycobacterium tuberculosis, fungal infections, cysticercosis, HTLV-1, and malignant external necrotizing otitis due to Pseudomonas. Similarly, it has been described in relation to autoimmune disorders, such as Wegener granulomatosis, rheumatoid arthritis, sarcoidosis, Behçet disease, Sjögren syndrome, and temporal arteritis. Finally, it can be related to neoplasia, such as carcinomatosis, lymphoma, and meningioma en plaque [5,9].

IgG4-related sclerosing disease was initially recognized as a form of autoimmune pancreatitis. However, it is now known that this disease can affect the bile duct, gallbladder, salivary glands, retroperitoneum, kidneys, lungs, and even, prostate. Pathologically, this disease is characterized by extensive infiltration of IgG4-positive cells in various organs [1]. Clinically, it often presents with mass-like lesions. Central nervous system involvement is rarely reported; however, recently it has been suggested that IgG4-related sclerosing disease represents a subset of cases previously diagnosed as idiopathic hypertrophic pachymeningitis [2-4]. IgG4-related sclerosing pachymeningitis may present a diffuse infiltrative pattern with or without mass formation [2-4]. To our knowledge, this is the first reported case of localized IgG4-related pachymeningitis with concomitant skull hyperostosis. Noda D et al. previously reported a case of idiopathic pachymeningitis with skull hyperostosis [10] but without clarifying its possible etiology.

Meningeal biopsies are frequently performed for a differential diagnosis of pachymeningitis, especially for those with infectious etiology [5,7,9]. In our case, surgical excision was performed to provide possible symptomatic relief as well as for diagnosis. In our case, thickening of the arachnoid membrane was left untouched to avoid parenchymal injury.

Although it has been reported that pachymeningitis responds well to steroid treatment and other immunosuppressants, we did not prescribe the above medications owing to concerns about the adverse effects of their long-term use [3,9,11,12]. In addition, this case was found only to be involved locally in dura and skull at admission, and without involvement of other organs, such as: lacrimal glands, salivary glands, or pancreas after detailed examinations.

The patient’s symptoms disappeared following surgical treatment. Thus, surgical excision to decrease the affected region may benefit patients with IgG4-related pachymeningitis, especially for patients with a solitary mass and without other organ involvement. Beyond our expectation, the infiltrative pattern of the involved arachnoid membrane disappeared following surgery. The self-limiting course may be related to the decrease in systemic IgG4 levels. It will be important to follow-up the patient for signs of recurrence and further organ involvement in the future.

## Conclusions

Despite its extremely rarity, IgG4-related sclerosing disease may present as a solitary intracranial condition. Surgical biopsy may be the only means of obtaining a differential diagnosis. Extensive excision of the intracranial lesion may be performed for symptomatic patients.

## Consent

Written informed consent was obtained from the patient for publication of this case report and any accompanying images.

A copy of the written consent is available for review by the Editor of this journal.

## Abbreviations

IgG4: Immunoglobulin G4; CSF: Cerebrospinal fluid; ABC: Avidin-biotin-complex.

## Competing interests

The authors declare that they have no competing interests.

## Authors’ contributions

C.K.L: Collections of the clinical data of the case and writing of the manuscript. D.M.L: Instructions and responsibility for the completion of this manuscript. Both authors read and approved the final manuscript.

## Pre-publication history

The pre-publication history for this paper can be accessed here:

http://www.biomedcentral.com/1471-2482/13/37/prepub
